# Microorganisms of Two Thermal Pools on Kunashir Island, Russia

**DOI:** 10.3390/biology10090924

**Published:** 2021-09-16

**Authors:** Aleksei S. Rozanov, Anton V. Korzhuk, Sergei V. Shekhovtsov, Gennady V. Vasiliev, Sergei E. Peltek

**Affiliations:** 1 Institute of Cytology and Genetics of Siberian Branch of Russian Academy of Sciences (ICG SB RAS), 630090 Novosibirsk, Russia; shekhovtsov@bionet.nsc.ru (S.V.S.); genn@bionet.nsc.ru (G.V.V.); peltek@bionet.nsc.ru (S.E.P.); 2 Faculty of Natural Sciences, Novosibirsk State University, 630090 Novosibirsk, Russia

**Keywords:** metagenome, hot spring, microbial community, genome, Kuril Islands

## Abstract

**Simple Summary:**

The Kuril Islands are a part of the Circum-Pacific Belt of volcanoes and have many hot springs. Nonetheless, due to the border regime, these islands are difficult to access, and microbial communities of the geothermal springs of these islands have hardly been studied microbiologically and have not been studied metagenomically at all. Here we conducted the first metagenomic study on two thermophilic microbial communities of Kunashir Island. Faust Lake is hot (48 °C) and highly acidic (pH 2.0), whereas the Tretyakovsky Thermal Spring is also hot (52 °C) but weakly acidic (pH 6.0). We demonstrated that water pH affects the composition of the microbial communities.

**Abstract:**

The Kuril Archipelago is a part of the Circum-Pacific Belt (Ring of Fire). These islands have numerous thermal springs. There are very few studies on these microbial communities, and none of them have been conducted by modern molecular biological methods. Here we performed the first metagenomic study on two thermophilic microbial communities of Kunashir Island. Faust Lake is hot (48 °C) and highly acidic (pH 2.0). We constructed 28 metagenome-assembled genomes as well as 17 16S ribosomal RNA sequences. We found that bottom sediments of Faust Lake are dominated by a single species of red algae belonging to the Cyanidiaceae family. Archaeans in Faust Lake are more diverse than bacteria but less abundant. The Tretyakovsky Thermal Spring is also hot (52 °C) but only weakly acidic (pH 6.0). It has much higher microbial diversity (233 metagenome-assembled genomes; 93 16S ribosomal RNAs) and is dominated by bacteria, with only several archaeans and one fungus. Despite their geographic proximity, these two thermal springs were found to not share any species. A comparison of these two lakes with other thermal springs of the Circum-Pacific Belt revealed that only a few members of the communities are shared among different locations.

## 1. Introduction

Microorganisms, especially prokaryotes, are capable of living in diverse environments, which may strongly differ from those present on the surface of the Earth in such parameters as temperature, pH, redox potential, and availability of organic matter and light. Extremophiles living at high temperatures have turned out to be some of the most promising as sources of new enzymatic activities for molecular biology and biotechnology. Their taxonomic diversity is the second most important parameter: these communities are reported to contain not only new species but also phyla that have not been found at mesophilic sites. Microbial communities of hydrothermal springs include representatives of extremely diverse taxa belonging to all three domains of life.

Life in extreme environments, including hot springs, was discovered as early as 1903 [1,2], but only the advent of molecular biology has provided us with the methods to uncover the whole diversity of extremophiles. Metagenomics allows one to investigate taxonomic diversity and metabolic properties of communities without their cultivation. This is particularly relevant for thermophiles, which are notoriously resilient to cultivation in the laboratory. Many of those are known only by their DNA sequences [3]. They are intensively studied due to their peculiar chemical composition and high value for applied research [4].

Sites of terrestrial hydrothermal outlets are scattered throughout the world in volcanism zones. Hydrothermal vents stand out not only in terms of temperature and pH but also in terms of chemical composition of the water. These factors contribute to the formation of unique combinations of microbial communities.

The Circum-Pacific Belt (also known as the Ring of Fire) is the world’s largest stretch of a seismically active zone with many sites of hydrothermal outlets. The Circum-Pacific Belt includes vast territories of the West coasts of both Americas, the East coast of Eurasia, and Oceania. There are many studies on microbial communities of terrestrial hot springs in various parts of the Circum-Pacific Belt: the USA [5,6], South America [7,8], New Zealand [9], Indonesia [10], the Philippines [11], Japan [12], and Russia (the Kamchatka Peninsula: [13,14,15]). The Kuril Archipelago is a long chain of islands ca. 1200 km long, located between Kamchatka and the Japanese Islands. Many of the Kuril Islands harbor volcanic activity and outlets of thermal water. There is little research on the prokaryotes inhabiting the hot springs of the Kuril Islands [16,17], and none of their microbial communities have ever been characterized by metagenomic methods. Kunashir is one of the most interesting islands in terms of volcanic activity: the diversity of chemical elements in its thermal springs is among the highest in the world [18]. For this reason, one of the volcanoes has been named after D.I. Mendeleev, the discoverer of the periodic table of elements. This diversity makes the island a unique sanctuary for the conservation of a wide variety of thermal microbial communities.

Here we conducted the first metagenomic study of extremophilic microbial communities of Kunashir Island. We also compared them to communities of other thermal springs via data available in NCBI databases.

## 2. Materials and Methods

### 2.1. Description of the Study Area

**Figure 1:**
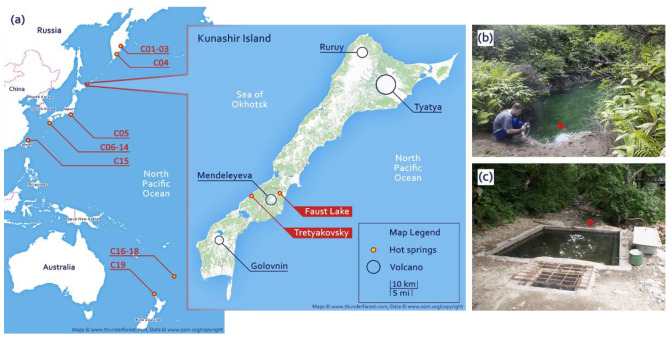
(**a**) The map of the locations of the investigated hot springs and (**b**,**c**) photographs of sampling sites: (**b**) Faust Lake (FAU) and (**c**) the Tretyakovsky Spring (TRT).

Faust Lake (FAU) is a small pool approximately 4 m in diameter located near the Pacific shore of Kunashir. Temperature at the sampling site (near the shore) was 48 °C and as high as 66 °C at the outlet. Faust Lake (Figure 1a,b) is very acidic with pH below 2.0. The Tretyakovsky Spring (TRT) (Figure 1a,c) is hot (52 °C) and weakly acidic (pH 6.0). We investigated the composition of microbial communities of these two lakes by next-generation sequencing. We collected samples of bottom sediments and extracted and sequenced total DNA. The obtained data were assembled into partial genomes and small subunit (SSU) ribosomal RNA (rRNA) sequences. To compare different hot springs of the Circum-Pacific Belt (Figure 1a), we compiled a dataset of metagenomic data from different locations.

### 2.2. Sample Collection and DNA Extraction

Sediment samples from FAU and TRT were collected into 50 mL Falcon tubes and fixed with an equal volume of distilled ethanol.

DNA isolation was performed with the NucleoSpin^®^ Soil kit (Macherey-Nagel Inc., Düren, Germany) according to the manufacturer’s protocol. Lysis was carried out using SL2 buffer in combination with the Enhancer SX additive.

### 2.3. Library Preparation and Sequencing

Sequencing libraries were prepared at the Genomic Research Center of the ICG SB RAS using the Nextera™ DNA Flex Library Prep Kit (Illumina, Inc., San Diego, CA, USA) according to the manufacturer’s instructions. The libraries were analyzed by means of 2100 Bioanalyzer. Sequencing was performed by the Center for Genetics and Reproductive Medicine GENETICO in Moscow on the Illumina NovaSeq 6000 platform (paired reads 100 bp long). The raw reads were deposited in the NCBI Sequence Read Archive (SRA) database under accession numbers SRR7903764 and SRR7903765.

### 2.4. Sequence Processing and Metagenomic Assembly

The sequence processing was carried out in the FastQC v.0.11.7 software [18] and Trimmomatic v.0.36 [19] with the following parameters: CROP, 97, and MINLEN, 40. The sequencing data extracted from the NCBI SRA were cleared of over-represented sequences (if present) and low-quality bases (options ILLUMINACLIP:<adapters.fa>:2:30:10 (if necessary), SLIDINGWINDOW: 6:15, TRAILING: 18, MINLEN: 40).

Adapter sequences for C17 data:

>TruSeq_Adapter_Index18

GATCGGAAGAGCACACGTCTGAACTCCAGTCACGTCCGCACATCTCGTATGCCGTCTTCTGCTTG

>PCR_Primer1_rc

AGATCGGAAGAGCGTCGTGTAGGGAAAGAGTGTAGATCTCGGTGGTCGCCGTATCATT

The samples were employed to assemble two metagenomes (one for FAU and one for TRT) using SPAdes v.3.11.1 [20] with the “—meta” option; other parameters were at their default values. Assembly quality was assessed in metaQUAST v.4.6.2 [21].

### 2.5. Metagenomic Binning

Extraction of draft metagenome-assembled genomes (MAGs) from metagenomic data was performed using MetaWRAP pipeline v.1.2.2 [22]. Clustering was performed by metaWRAP-binning and metaWRAP-bin_refinement modules that use metaBAT v.2.12.1 [23], CONCOCT v.1.0 [24] (both with default parameters), and MaxBin v.2.2.4 [25] (by means of a set of universal marker genes, option “-markerset 40”) for clustering and CheckM v.1.0.12 [26] for evaluating the quality of bins. These bins were then reassembled by the metaWRAP-reassemble_bins module (uses SPAdes version 3.13.0). Information on the size of the extracted genomes, GC composition, and N50 was obtained by means of QUAST v.5.0.2. Genome completeness and contamination levels were assessed in CheckM. The information on the abundance of genomes was calculated in the Salmon software v.1.0 [27] in copies of the genome per 1 million reads.

### 2.6. Taxonomic Analysis of MAGs

For this purpose, ribosomal protein sequences were extracted from each bin using FragGenScan v.1.30 [28] and HMMer v.3.1b1 [29], which were then utilized to search for matches in the “NCBI nr” database via blastp.

Phylogenetic trees of MAGs were built in PhyloPhlAn v.3.0.55 [30,31], using the standard phylophlan database with 400 universal marker genes, a supermatrix_aa.cfg configuration file, the “--accurate” option on, and the option “--diversity” set to “high.” Visualization was performed in GraPhlAn v.1.1.3 [32]. To construct the tree of life, 1003 reference genomes of bacterial and archaeal isolates were used [33].

### 2.7. Reconstruction of Full-Length 16S rRNA Sequences

To reconstruct the 16/18S rRNA sequences from the original reads, we employed PhyloFlash v.3.3b1 [34]. Phylogenetic trees were constructed by the maximum likelihood method in MEGA v.10.0.5 [35]. The substitutions model was selected via MEGA’s built-in best model search tool. A total of 500 bootstrap repetitions were performed. Bootstap values are given for branches with support greater than 70%.

### 2.8. Data on Microbial Communities of the Circum-Pacific Belt

To compare them with the microbial communities of the FAU and TRT geothermal springs, metagenomic data on the microbial communities of hot springs located in the Circum-Pacific Belt were extracted from the NCBI SRA database. The data were retrieved by location according to the following criteria: organism, hot springs metagenome; library strategy, WGS; library source, METAGENOMIC, and library layout, PAIRED. Four datasets were obtained from Kamchatka (C01–C04), 10 from Japan (C05–C14), one from Taiwan (C15), and four from New Zealand (C16–C19). The location of the hot springs included in the analysis is presented in Figure 1, and detailed information about the data is given in Table 1 and Table 2. The sequences of SSU rRNA and ribosomal proteins were obtained in the same way as the FAU and TRT data.

## 3. Results

### 3.1. Quality Filtering and Assembly

We obtained 206,089,215 reads for FAU and 421,795,959 for TRT. The reads had high quality (average of 36–38 on the phred-33 scale); however, abnormal-nucleotide occurrence was noted at the last positions of all reads. Furthermore, there was a small number of sequences of 35–39 bp in length among the reads. Therefore, three nucleotides at the end were deleted, and only sequences longer than 40 bp were kept. As a result of the processing, 1,235,736 (0.6% of the initial number of reads) and 148,563 (0.035% of the initial number of reads) reads were lost in the FAU and TRT datasets, respectively. Assembly by SPAdes yielded 20,806 contigs >1000 bp long for FAU and 279,624 contigs >1000 bp long for TRT. Details are given in Supplementary Table S1.

### 3.2. MAGs

We utilized MetaWRAP to assemble 26 draft MAGs for FAU and 233 for TRT with completeness >75% and contamination <5% (17 and 157 of those, respectively, are high-quality MAGs [36] with >90% completeness and <5% contamination) (Supplementary Tables S2 and S3). Two high-coverage genomes affiliated with a red alga and its chloroplast were separately extracted from the FAU assembly using MaxBin v.2.2.4. For FAU, these 26 + 2 MAGs include 65.7% of the nucleotides present in the total assembly; for TRT, the 233 MAGs make up 56% of the assembly. Information about the obtained MAGs is provided in the Supplements (Supplementary Tables S2 and S3).

The majority of the assembled genomes from FAU belong to Archaea (a total of 14): 11 to Euryarchaeota; one each to Candidatus Korarchaeota and Candidatus Parvarchaeota, and one could not be classified. Twelve of the genomes were bacterial: eight from Actinobacteria, three from Proteobacteria, and one from Firmicutes. We also assembled a nuclear and a plastid genome for one red alga.

The majority of MAGs from TRT (231 out of 233) were found to belong to Bacteria (Table 3). Proteobacteria were the dominant group, with 73 assembled genomes (Alphaproteobacteria: 27; Betaproteobacteria: 19; Gammaproteobacteria: 5; Deltaproteobacteria: 12; Epsilonproteobacteria: 2; Hydrogenophilalia: 2; Oligoflexia: 1, and Unclassified: 5). The second most abundant type was Bacteroidetes with 20 MAGs, followed by Planctomycetes with 13 MAGs. The details on the detected MAGs are listed in Table 3.

Only two MAGs are affiliated with Archaea, one each with Euryarchaeota and Candidatus Woesearchaeota. In contrast to FAU, no eukaryotes were detected. On the other hand, 13 of the obtained metagenomes belong to photosynthetic bacteria: nine to Cyanobacteria, and four to Chloroflexi (Table 3). Cyanobacteria were represented by (Supplementary Table S2: MAG IDs TRT-7, TRT-8, TRT-16, TRT-74, TRT-131, TRT-183, TRT-205, TRT-209, and TRT-242): *Gloeocapsa* sp. PCC 7428, *Stanieria* sp. NIES-3757, and seven unclassified; Chloroflexi were represented by (Supplementary Table S2: MAG IDs TRT-27, TRT-45, TRT-132, and TRT-144): *Chloroflexus islandicus*, *Roseiflexus* sp. RS-1, an Oscillochloridaceae bacterium, and a Chloroflexales bacterium.

**Figure 2:**
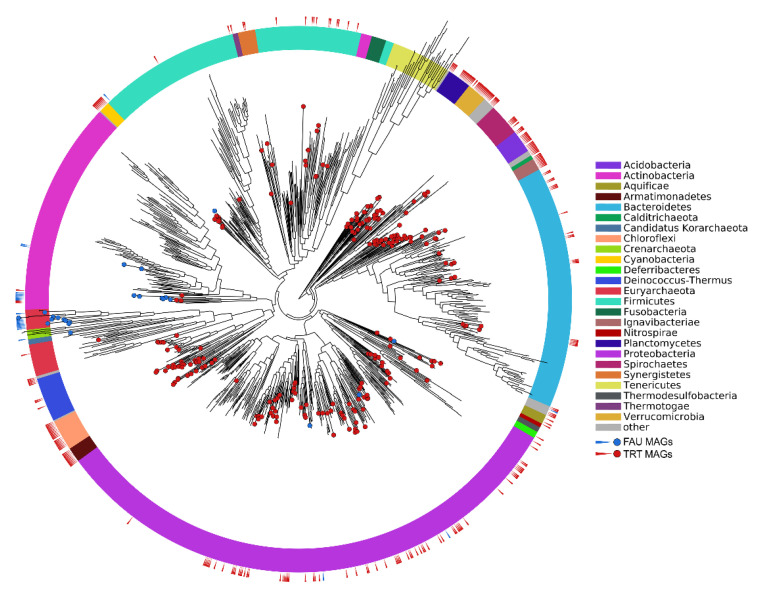
Phylogenetic position of the obtained MAGs on the prokaryotic tree of life. Colors of the ring sectors denote microbial phyla.

The phylogenetic position of the obtained MAGs on the tree of life is shown in Figure 2.

**Figure 3:**
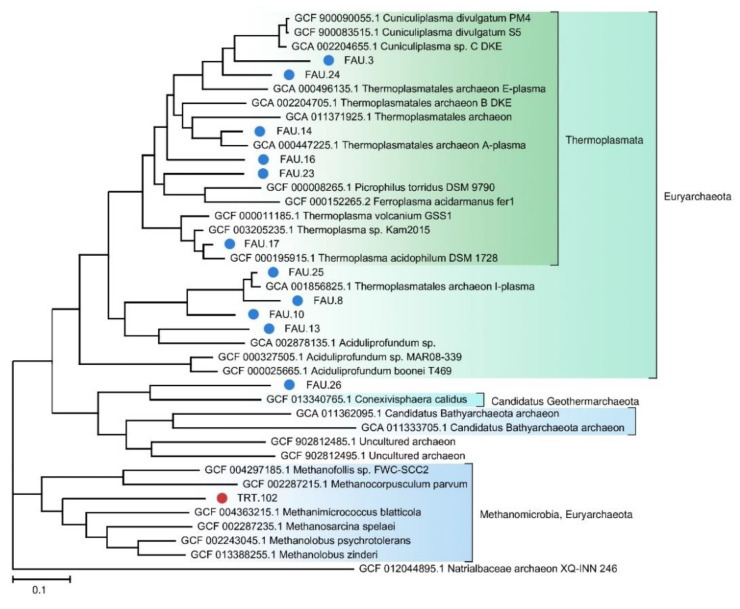
The phylogenetic tree of MAGs affiliated with the Archaea domain. FAU MAGs No. 6, 11, and 18 and TRT MAGs No. 240 and 159 were not placed on the tree because they do not contain enough marker sequences.

We also constructed a separate phylogenetic tree for archaeal MAGs (Figure 3).

### 3.3. SSU rRNA Sequences

The assembly made by SPAdes yielded few SSU rRNA gene sequences. To obtain more information on rRNA sequences, we ran a search by means of PhyloFlash. In the FAU dataset, 426,217 (~0.208% of the total) paired reads related to 16/18S rRNA were identified; 46.2% of them were assembled into 17 sequences >1 kbp long: five from Euryarchaeota; two from Proteobacteria; one each from Actinobacteria, Firmicutes, Thermotogae, and Candidatus Saccharibacteria, and three unclassified archaeal sequences. We also found the 18S rRNA sequence for the red algae and 16S rRNA genes for its plastid and mitochondria.

In the TRT dataset, 276,374 paired reads (~0.066% of the total) were identified as 16/18S rRNA gene sequences. Of these, 26.3% were assembled into 93 sequences over 1 kbp long: 19 from Proteobacteria; 10 from Chloroflexi; 10 from Bacteroidetes; 9 from Firmicutes; 4 from Cyanobacteria; 3 from Planctomycetes; 4 from Acidobacteria; 2 from Verrucomicrobia; 5 from Spirochaetes; 3 from Deinococcus–Thermus; 2 from Synergistetes; 2 from Amorphea; 2 from Armatimonadetes; 2 from Aquificae; 1 from Actinobacteria; 1 from Euryarchaeota; 1 from Thermotogae; 1 from Patescibacteria; 1 from Nitrospirae; 1 from Nanoarchaeota; 1 from Elusimicrobia; 1 from Deferribacteres; 1 from Crenarchaeota; 1 from Sercytochromatia; 1 from Sumercytochromatia; 1 from Candidatus Melainabacteria; 1 from Candidatus Hydrothermae; 1 from Candidatus Bipolaricaulota; 1 from Calditrichaeota, as well as a eukaryotic 18S sequence from Rotifera and a mitochondrial 18S sequence of *Acanthamoeba* sp.

The taxonomic data compared with the Silva database and SSU rRNA gene sequences are shown in Supplementary Table S3.

**Figure 4:**
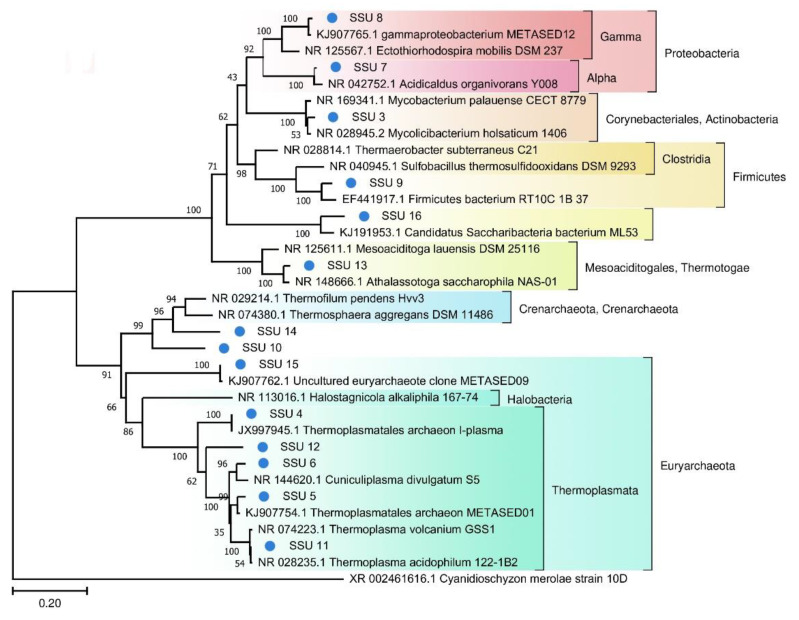
The phylogenetic tree of the prokaryotic SSU sequences from FAU and their closest homologs (The Tamura–Nei substitution model with the gamma distribution (TN93 + G)).

A phylogenetic tree of prokaryotic SSU rRNA sequences is depicted in Figure 4. Most of the SSU rRNA gene sequences from the FAU microbial community are phylogenetically distant from known species.

**Figure 5:**
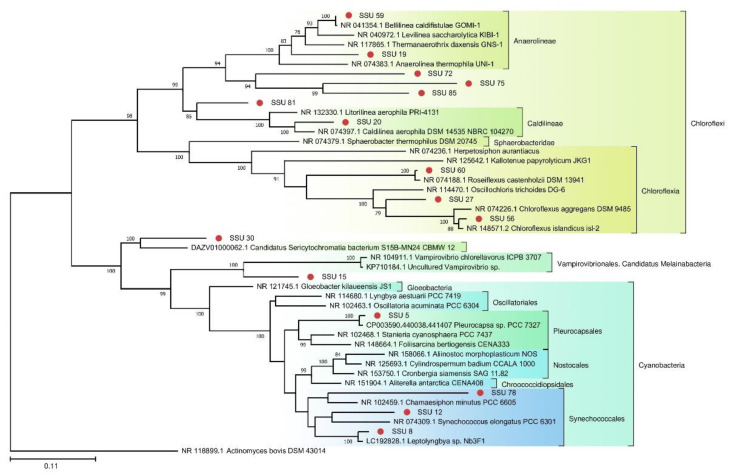
The phylogenetic tree of SSU rRNA sequences of photosynthetic representatives of the TRT community and their closest homologs from the NCBI nt database (The Tamura–Nei substitution model with gamma distribution and invariant sites (TN93 + G + I)).

**Figure 6:**
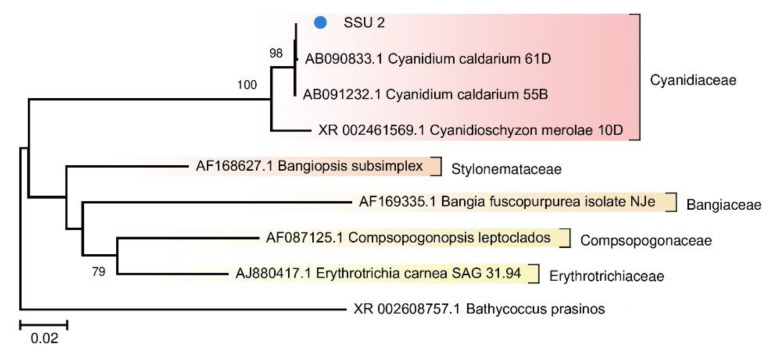
The phylogenetic tree of the SSU rRNA sequence of the red alga from the FAU community and other representatives of Rhodophyta from the NCBI nt database (The Tamura–Nei substitution model with gamma distribution and invariant sites (TN93 + G + I)).

Because photosynthetic microorganisms are of particular interest as the basis of the food chain, we built a separate tree using sequences of photosynthetic bacteria from the TRT microbial community (Figure 5). A tree was also constructed for the red algae from FAU (Figure 6).

**Figure 7:**
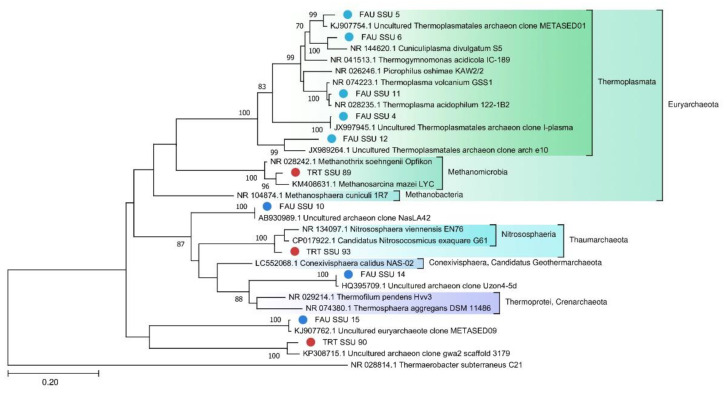
The phylogenetic tree of SSU rRNA belonging to the Archaea domain (The general time reversible model with gamma distribution (GTR + G)).

A phylogenetic tree of archaeal SSU rRNA sequences is depicted in Figure 7.

### 3.4. Taxonomic Composition of Microbial Communities According to SSU rRNA Data

**Figure 8:**
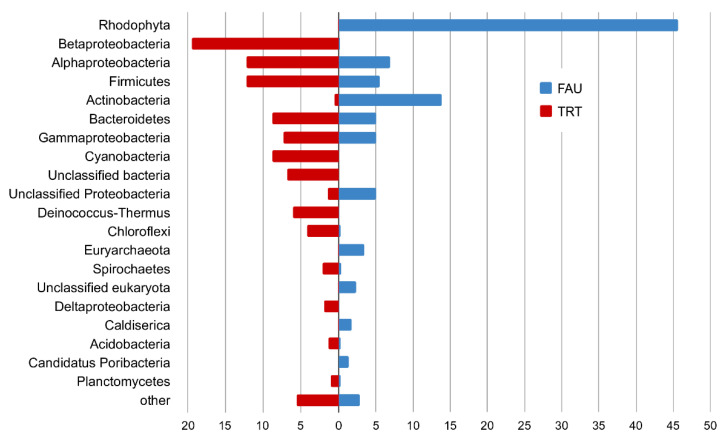
The plot comparing phyla abundance (classes in the case of Proteobacteria) between FAU and TRT (%).

In the PhyloFlash software, the reads mapped to the SSU rRNA gene were extracted, and their taxonomic position was determined. A comparison of abundance levels of phyla and classes of Proteobacteria between the studied communities according to the analysis of reads related to SSU rRNA is presented in Figure 8.

Most of the SSU rRNA reads from FAU were assigned to chloroplast and mitochondrial genomes; in total, they constituted 86% of all rRNA sequences. These reads were excluded from the analysis of taxonomic diversity.

In the remaining set of reads, the SSU rRNA gene of the red alga *Cyanidium* accounted for 45.7 and 47.6% of reads that belonged to bacteria. This group was the most diverse: among bacteria, most reads were from Proteobacteria (17.2%), mostly Alphaproteobacteria (7.0%), Gammaproteobacteria (5.0%), and unassigned proteobacteria (5.0%). Actinobacteria were the second most abundant phylum, represented mostly by Mycobacterium (9.7%) and Acidimicrobiia (1.2%). Firmicutes (mostly Clostridia) accounted for 5.5%, and Bacteroidetes for 5.0%. Finally, 1.7% of reads were assigned to Caldiserica, and 1.4% to Candidatus Poribacteria.

Archeal SSU reads constituted 4.1% of the FAU data. They were mainly represented by Euryarchaeota (3.4%), Micrarchaeota (0.3%), and Crenarchaeota (0.3%).

The complete list of taxa according to the SSU analysis is given in Supplementary Table S4.

In the TRT dataset, Archaea account only for 0.27% of reads, and eukaryotes for 0.15%. Among the bacterial phyla, the most abundant ones were Betaproteobacteria (19.5%), Alphaproteobacteria (12.2%), Firmicutes (12.2%), Cyanobacteria (8.8%), Bacteroidota (8.8%), Deinococcus–Thermus (6.1%), Chloroflexi (4.2%), Spirochaetes (2.1%), Deltaproteobacteria (1.9%), Acidobacteria (1.3%), and Planctomycetes (1.0%) (See Supplementary Table S4 for a complete list).

### 3.5. Analysis of Geothermal Microbial Communities of the Circum-Pacific Belt

For the analysis of the strains, we recovered SSU rRNA sequences from metagenomic data in open repositories (Table 2). Extraction of SSU rRNA was performed as described in Section 2.7.

According to the SSU rRNA data (Supplementary Table S7), one strain from FAU shared 99% sequence similarity with a strain from Cub Bath (C19; New Zealand); two strains >97%, and another two >95%. Cub Bath is somewhat colder (24.5–33.4 °C) and less acidic (pH 3.2–3.6) as compared to FAU. One strain from Arkashin Schurf (C01; Russia, Kamchatka) and one from Shi-Huang-Ping (C15; Taiwan) also proved to be relatively closely related to strains from FAU but with low coverage. We should point out that we failed to detect any rRNAs in four out of the 19 assembled metagenomes, and in the other two, we found only one rRNA sequence. Therefore, we also compared ribosomal proteins from these metagenomes, from FAU, and from TRT (Supplementary Table S8). Strains from FAU have relatives at Mutnovsky volcano (C04; Russia, Kamchatka) and Ioudani (C13; Japan). Both C04 and C13 are hot (70–88 °C) and acidic (pH 3–4).

SSU rRNA sequences >99% similar to strains from TRT were found in Zavarzin (C03; Russia, Kamchatka), Nonoykoya (C09; Japan), and Kinyu (C10; Japan) (Supplementary Table S7). An analysis of ribosomal proteins revealed related strains in Kamchatka [Arkashin Schurf (C01) and Mutnovsky (C04)] and Japan [Jinata Onsen Pool (C05), Nonoykoya (C09), Kinyu (C10), Eastern Pool (C17), and Green Lake (C18)].

**Figure 9:**
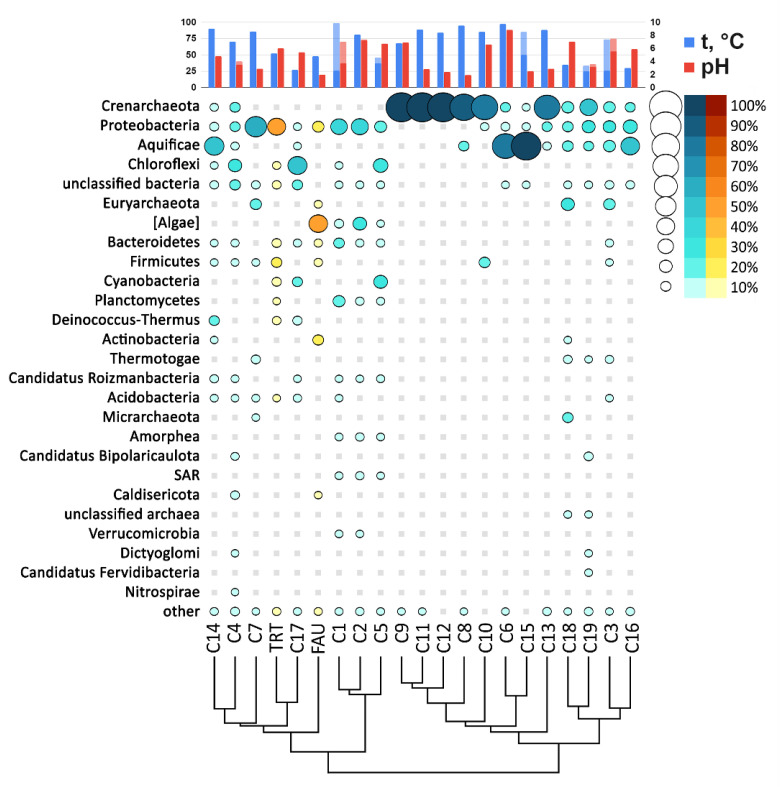
The abundance bubble chart of microbial phyla in the FAU, TRT (yellow), and C01–C19 (blue) communities.

To compare the taxonomic diversity at the phylum level, we constructed an abundance chart for different springs along the Circum-Pacific Belt taking into account their pH and temperature (Figure 9).

## 4. Discussion

In this work, we obtained the first metagenomic data on thermal springs of the Kuril Islands. The two analyzed springs were found to have different chemical characteristics, which are probably the reason for the observed differences between their microbial communities. TRT has weakly acidic pH and is characterized by high diversity (Shannon index 4.4). In contrast, the highly acidic pH of FAU is probably the cause of its low diversity (Shannon index 1.7).

In the course of analyzing metagenomic data, we obtained 259 prokaryotic MAGs (26 for FAU and 233 for TRT). The extracted TRT MAGs belong to a wide range of phyla, while FAU was found to have significantly lower phylogenetic diversity. Most of the MAGs have close relatives among the known bacteria and archaea. Nevertheless, some of the genomes from FAU differ significantly from the currently known sequences.

Moreover, FAU has lower species diversity: the algal genome represents 45.7% of all sequences, and another 9.7% are affiliated with a *Mycobacterium*. Most of the species diversity in FAU is represented by extremophilic Archaea from recently discovered phyla. It is noteworthy that most of them proved to be only remotely related to congeneric species.

Shannon indices, which reflect the complexity of microbial communities, differ dramatically between the studied lakes. FAU has a Shannon index of ~1.4, which is very low and typical for lithotrophic communities of extremely acidic springs of high or moderate temperatures. In contrast, the Shannon index of TRT is 4.4. This is quite high and close to the values of soil microbial communities. Thus, 50 °C is not a serious impediment for the existence of complex prokaryotic communities, whereas pH < 2 is [37,38,39]. Low species diversity under acidic conditions is due to basic physical principles. The hydrogen ion easily penetrates through the cell membrane into the cytoplasm, and it takes a lot of energy to remove it. As a result, minimization of energy expenditure by the cell for other needs, including the maintenance of the genome, becomes vital for survival.

### 4.1. Metabolism of Microbial Communities of FAU and TRT

Photosynthetic organisms are the basis of most communities, including those of geothermal springs. Environmental pH is often critical for photosynthetic microorganisms. Prokaryotes, especially photosynthesizing ones, are poorly adapted to life in low-pH environments. Cyanobacteria thrive at pH levels close to neutral and especially well at high pH: 8.0–11.0. Massive, complicatedly organized microbial communities up to several tens of centimeters thick arise in the waters of springs having high pH [14]. No microbial mats were seen in TRT because the spring had been cleaned shortly before the sampling. Nevertheless, photosynthetic bacteria, both oxygenic (Cyanobacteria 8.8%) and anoxygenic (Chloroflexi 1%), were identified in this community (Figure 5). The presence of these species indicates high probability of the formation of microbial mats.

Photosynthetic microorganisms of FAU were found to be represented by a single eukaryote, an alga closely related to *Cyanidium caldarium* (Figure 6). It is a small primitive unicellular red alga that lives in sulfate-rich ultra-acidic hot springs. Eukaryotes have a smaller surface-to-volume ratio as compared to prokaryotes, which makes it easier for them to adapt to acidic conditions. According to the literature, this alga is an obligate autotroph, has a nucleus, mitochondria, and a large single chloroplast but does not contain vacuoles [37,40]. Due to unusual structure and ecological preferences, representatives of the genus *Cyanidium* were assigned to different taxa: cryptomonads, Cyanobacteria, and green algae. Currently, *Cyanidium* is considered the most primitive organism among red algae [38]. The coverage of its chloroplast genome was ~15-fold greater than that of the nuclear genome.

Our results are in good agreement with the available data on the presence of photosynthetic microorganisms in hot springs with different pH levels.

### 4.2. Archaeal Communities of FAU and TRT

Archaea constitute only 0.26% of the TRT microbial community, probably because the local environment is more suitable for bacteria. Nevertheless, two MAGs were assembled from the sequencing data: Euryarchaeota and Candidatus Woesearchaeota. On the contrary, archaea make up a significant part of the FAU community. Although more reads in FAU belong to Bacteria, archaea are more diverse in that spring. The majority of the detected archaeal strains proved to be more or less closely related to other known thermoacidophilic species. Eleven MAGs that together are the most abundant among archaeal sequences belong to Euryarchaeota. Ten MAGs fall within various branches of Thermoplasmata. Thermoplasmata are ubiquitous in acidic thermal and mesophilic environments [39]. Several MAGs were found to be affiliated with poorly studied archaeal phyla. For example, one of them is closely related to the Candidatus *Conexivisphaera calidus* (Candidatus *Geothermarchaeota* phylum) that inhabits Iceland hot springs with temperatures over 70 °C. MAG No. 18 turned out to be a relative of Candidatus *Parvarchaeum acidophilus* from the Parvarcheota group, and MAG No. 11 a relative of Candidatus Korarchaeota archaeon.

To assess the phylogenetic position of archaea, we constructed trees for MAGS (Figure 7) and 16S rRNA (Figure 8).

Significant distances between the sequences of the obtained genomes and those retrieved from databases indicate that they belong to poorly studied phyla. On the other hand, on the tree (Figure 8) constructed for the archaea SSU rRNA gene sequences, one can see that they are quite close to known species or sequences from metagenomic data.

### 4.3. The Comparison with Other Thermal Springs along the Circum-Pacific Belt

Metagenomic data enable one to directly compare the composition of microbial communities and relative abundance levels of individual microorganisms. This approach overcomes the biases introduced by cultivation-based and PCR-based methods and therefore can be currently considered the most accurate technique in this respect. Nonetheless, there are still not many data on individual thermal springs. For this study, we assembled a set of 19 metagenomes of terrestrial hot springs from various regions of the Old-World part of the Circum-Pacific Belt: the Kamchatka Peninsula (Russia), the Japanese Islands, Taiwan, and New Zealand (Table 1). Temperature and pH of these springs vary widely. We noticed that FAU shares no strains with TRT, despite their close proximity, and very few species with other thermal springs. Related strains were detectable in acidic (pH 2.5–4.0) and warm to moderately hot springs. A group of acidic (pH 1.9–2.9) and very hot (>84 °C) springs in Japan shares no species with FAU, probably indicating that extreme temperature plays a prohibitive role in species composition.

As compared to FAU, TRT shares much more microbial species with other springs in the above dataset. The most closely related water bodies are Jinata Onsen Pool 3 (Japan; 18 shared species), two thermal springs on Raoul Island (New Zealand; 11 and 10 shared species), and Nonoykoya (Japan; 10 shared species). As expected, the Jinata Onsen pool has similar water parameters (pH 6.7 and 37.3–46.0 °C). Nevertheless, because over 300 genomes were assembled from TRT, we can estimate that its overlap in microbial composition is only ~5% at most.

Microbial diversity is generally thought to follow the principle of Baas Becking (1934): “Everything is everywhere: but the environment selects,” although there are numerous exceptions to this rule [41]. The comparison of microbial communities of various thermal springs along the Circum-Pacific Belt suggests that certain microbial strains with identical marker sequences are indeed found tens of thousands of kilometers apart. Nonetheless, the composition of the communities appears to be highly specific despite close temperature and pH parameters.

According to relative abundance of various phyla (Figure 9), TRT is most closely related to a spring on Raoul island. FAU is close to these two springs too, as well as to hydrothermal water bodies from America. Thus, the studied hot springs of Kunashir Island are closer in their composition to geographically distant rather than closer water bodies.

## 5. Conclusions

In this work, we performed the first metagenomic analysis of thermal springs of Kunashir Island. This is the first step in closing the gap in such studies on the Kuril Islands. Microbial communities of thermal springs have been extensively studied for several decades all over the world. Nonetheless, we find that most microorganisms in the analyzed lakes are new to science. This observation suggests that we have only begun to unveil the wealth of new microbial taxa living in hot springs and indicates potential usefulness of the thermal lakes on the Kuril Islands as a source of new strains and enzymes for biotechnology.

This paper presents the first metagenomic analysis of two hot springs located on Kunashir Island. This work partially closes the gap in the knowledge about microbial communities of geothermal springs on the Kuril Islands. The investigated springs are characterized by a moderately high temperature of ~50 °C and are substantially different in pH. The Tretyakovsky Spring has neutral pH, whereas Faust Lake features strongly acidic pH, <2. The springs are located on the slopes of D.I. Mendeleev volcano, which is known for the highest chemical diversity of geothermal waters, including the presence of rare earth elements.

Our assessment of biological diversity revealed that the prokaryotic community of the Tretyakovsky Spring is composed of a large number of bacterial species. The complexity of its organization is close to that of the surface ecosystems that have moderate conditions. The microbial community of Faust Lake turned out to be very poor, and the average genome size is <2 mbp. The simplicity of this microbial community’s organization and the small size of genomes are related to high consumption of energy needed for cell homeostasis under acidic conditions.

The obtained metagenomic data were compared with those available in open databases documenting similar microbial communities of the Circum-Pacific Belt. We demonstrated that the microbial communities of the springs under study are unique and significantly different in taxonomic composition from microbial communities of other parts of the Circum-Pacific Belt. FAU and TRT contain many microbes that can be classified as new species. This finding may be explained by the exceptional chemical composition of waters in the two springs. This is especially true for the microbial community of Faust Lake. Accordingly, we hope that new enzymes can be found there whose unusual sequences may be useful to the scientific community for solving biotechnological problems.

## Figures and Tables

**Table 1:** Information about hot springs.

Sample ID	Hot Spring Name	Location	Coordinates (N, E)	t, °C	pH
C01	Arkashin Schurf	Russia: Kamchatka, Uzon Caldera	54.5, 160.005556	26–99	3.7–7.0
C02	-	Russia: Kamchatka, Uzon Caldera	54.5, 159.967	81	7.2–7.4
C03	Zavarzin	Russia: Kamchatka, Uzon Caldera	54.498056, 160.014444	26–74	5.5–7.5
C04	-	Russia: Kamchatka, Mutnovsky volcano	52.453, 158.195	70	3.5–4.0
C05	Jinata Onsen Pool	Japan: Tokyo Prefecture, Shikinejima Island	34.318, 139.216	37.3–46	6.7
C06	Miyama	Japan: Kagoshima Prefecture, Kirishima	31.57, 130.46	96.8	8.8
C07	Ginyu	Japan: Kagoshima Prefecture, Kirishima	31.55, 130.48	85.5	2.9
C08	Kurinodake	Japan: Kagoshima Prefecture, Kirishima	31.55, 130.47	94.7	1.9
C09	Nonoykoya	Japan: Kagoshima Prefecture, Kirishima	31.55, 130.47	68	6.9
C10	Kinyu	Japan: Kagoshima Prefecture, Kirishima	31.55, 130.47	84.5	6.6
C11	Nonoyufunki	Japan: Kagoshima Prefecture, Kirishima	31.54, 130.49	89.1	2.8
C12	Torigigoku	Japan: Kagoshima Prefecture, Kirishima	31.54, 130.49	84	2.4
C13	Ioudani	Japan: Kagoshima Prefecture, Kirishima	31.53, 130.5	88	2.9
C14	Yamanoshiro	Japan: Kagoshima Prefecture, Kirishima	31.53, 130.49	90.4	4.8
C15	Shi-Huang-Ping	Taiwan: New Taipei City	25.19544, 121.60245	50–85	2.5
C16	Marker Bay Pool	New Zealand: Raoul Island	−29.26238, −177.92085	29.9	5.9
C17	Eastern Pool	New Zealand: Raoul Island	−29.26238, −177.919075	27.2	5.43
C18	Green Lake	New Zealand: Raoul Island	−29.263502, −177.920697	34.5	6.96
C19	Cub Bath	New Zealand: Northland, Tiger Springs area of Ngawha Springs	−35.407515, 173.8554755	24.5–33.4	3.2–3.6

**Table 2:** Metagenomic data extracted from the NCBI SRA and information on the number of extracted reads and assembled SSU sequences.

Sample ID	SRA Accession Number	Read Length	# of Reads	SSU rRNA Read Pairs	Fraction Assembled	# of Assembled SSU
C01	SRR6344961	84	26,454,313	12,113	8.586%	8
C02	SRR6311222	112	12,749,852	10,060	20.101%	9
C03	SRR6344962	84	29,167,348	18,223	5.743%	10
C04	SRR6311221	112	7,127,384	992	0.0%	0
C05	SRR7905025	101	34,244,964	30,374	33.900%	22
C06	DRR163688	201	4,991,937	3856	0.0%	0
C07	DRR163692	251	16,806,151	8678	29.454%	4
C08	DRR163689	101	58,832,615	16,668	0.0%	0
C09	DRR163687	101	59,857,660	55,170	19.073%	20
C10	DRR163685	101	59,915,181	45,654	28.130%	6
C11	DRR163693	201	19,757,761	15,274	10.355%	5
C12	DRR163691	251	5,922,448	3063	6.468%	1
C13	DRR163690	101	59,217,452	17,955	0.0%	0
C14	DRR163686	101	55,210,615	111,457	28.017%	1
C15	SRR1297203	101	23,280,105	35,097	36.431%	8
C16	SRR10063241	150	48,525,851	22,116	28.861%	55
C17	SRR10063242	150	43,957,771	22,466	28.272%	46
C18	SRR10063240	150	49,791,620	33,157	36.758%	54
C19	SRR11457942	100	23,179,480	26,340	33.101%	17

**Table 3:** The number of MAGs with completeness > 75%, contamination <5%, assembled 16S rRNAs >1 kbp long, and with identity >90% obtained for FAU and TRT.

Phylum	FAU MAGs	FAU SSU rRNA	TRT MAGs	TRT SSU rRNA
Acidobacteria	0	0	13	4
Actinobacteria	8	1	3	1
Amorphea	0	0	0	2
Aquificae	0	0	2	2
Armatimonadetes	0	0	11	2
Bacteroidetes	0	0	20	10
Calditrichaeota	0	0	1	1
Chloroflexi	0	0	19	10
Chloroplast	1	1	0	0
Cyanobacteria	0	0	9	4
Deferribacteres	0	0	1	1
Deinococcus–Thermus	0	0	6	3
Elusimicrobia	0	0	1	1
Euryarchaeota	11	5	1	1
Firmicutes	1	1	13	9
Gemmatimonadetes	0	0	3	0
Ignavibacteriae	0	0	8	0
Mitochondria	0	1	0	1
Nitrospinae	0	0	1	0
Nitrospirae	0	0	2	1
Planctomycetes	0	0	13	3
Proteobacteria	3	2	73	19
Rhodophyta	1	1	0	0
Spirochaetes	0	0	7	5
Synergistetes	0	0	2	2
Thaumarchaeota	0	0	0	1
Thermotogae	0	1	1	1
Verrucomicrobia	0	0	13	2
Candidate division WOR-3	0	0	1	0
Candidatus Bipolaricaulota	0	0	2	1
Candidatus Hydrothermae	0	0	0	1
Candidatus Korarchaeota	1	0	0	0
Candidatus Margulisbacteria	0	0	0	1
Cyanobacteria/Melainabacteria group (phylum Candidatus Melainabacteria)	0	0	1	1
Cyanobacteria/Melainabacteria group (class Candidatus Sericytochromatia)	0	0	0	1
Candidatus Parvarchaeota	1	0	0	0
Candidatus Riflebacteria	0	0	1	0
Candidatus Roizmanbacteria	0	0	0	1
Candidatus Woesearchaeota	0	0	1	0
Candidatus Saccharibacteria	0	1	0	0
unclassified Bacteria	0	0	4	0
unclassified Archaea	1	3	0	1

## Data Availability

FAU SRA: https://www.ncbi.nlm.nih.gov/sra/SRR7903764 (accessed on 22 August 2019), TRT SRA: https://www.ncbi.nlm.nih.gov/sra/SRR7903765 (accessed on 1 October 2019).

## Supplementary Materials

The following are available online at https://www.mdpi.com/article/10.3390/biology10090924/s1: Table S1: Assembly statistics for FAU and TRT metagenomes; Table S2: Assembly statistics and taxonomy for each MAG; Table S3: Statistics and taxonomy (SILVA) of the recovered SSU rRNA gene sequences from FAU and TRT; Table S4: Statistics regarding the taxonomy based on the analysis gene SSU rRNA reads; Table S5: Assembly statistics for C01–C19 metagenomes; Table S6: Statistics and taxonomy (SILVA) of the recovered SSU rRNA gene sequences from C01–C19; Table S7: Comparison of SSU rRNA sequences from FAU and TRT with SSU rRNA sequences from C01–C19; Table S8: Comparison of sequences of genes—coding for ribosomal proteins extracted from MAGs of FAU and TRT—with sequences of metagenomes from C01–C19.

## References

[1] Setchell W.A. (1903). The Upper Temperature Limits of Life. Science.

[2] Brock T.D. (1967). Life at high temperatures. Evolutionary, ecological, and biochemical significance of organisms living in hot springs is discussed. Science.

[3] Zaremba-Niedzwiedzka K., Caceres E.F., Saw J.H., Bäckström D., Juzokaite L., Vancaester E., Seitz K.W., Anantharaman K., Starnawski P., Kjeldsen K.U. (2017). Asgard archaea illuminate the origin of eukaryotic cellular complexity. Nature.

[4] Shetty R., Vestergaard M., Jessen F., Hägglund P., Knorr V., Koehler P., Prakash H.S., Hobley T.J. (2017). Discovery, cloning and characterisation of proline specific prolyl endopeptidase, a gluten degrading thermo-stable enzyme from Sphaerobacter thermophiles. Enzym. Microb. Technol..

[5] Wilson M.S., Siering P.L., White C.L., Hauser M.E., Bartles A.N. (2008). Novel Archaea and Bacteria Dominate Stable Microbial Communities in North America’s Largest Hot Spring. Microb. Ecol..

[6] Siering P.L., Wolfe G.V., Wilson M.S., Yip A.N., Carey C.M., Wardman C.D., Shapiro R.S., Stedman K.M., Kyle J., Yuan T. (2013). Microbial biogeochemistry of Boiling Springs Lake: A physically dynamic, oligotrophic, low-pH geothermal ecosystem. Geobiology.

[7] Urbieta M.S., Toril E.G., Alejandra Giaveno M., Bazán Á.A., Donati E.R. (2014). Archaeal and bacterial diversity in five different hydrothermal ponds in the Copahue region in Argentina. Syst. Appl. Microbiol..

[8] Sofía Urbieta M., González-Toril E., Aguilera Á., María B., Giaveno A., Donati E. (2015). Comparison of the microbial communities of hot springs waters and the microbial biofilms in the acidic geothermal area of Copahue (Neuquén, Argentina). Extremophiles.

[9] Stewart L.C., Stucker V.K., Stott M.B., de Ronde C.E.J. (2018). Marine-influenced microbial communities inhabit terrestrial hot springs on a remote island volcano. Extremophiles.

[10] Baker G.C., Gaffar S., Cowan D.A., Suharto A.R. (2001). Bacterial community analysis of Indonesian hot springs. FEMS Microbiol. Lett..

[11] Huang Q., Jiang H., Briggs B.R., Wang S., Hou W., Li G., Wu G., Solis R., Arcilla C.A., Abrajano T. (2013). Archaeal and bacterial diversity in acidic to circumneutral hot springs in the Philippines. FEMS Microbiol. Ecol..

[12] Martinez J.N., Nishihara A., Lichtenberg M., Trampe E., Kawai S., Tank M., Kühl M., Hanada S., Thiel V. (2019). Vertical Distribution and Diversity of Phototrophic Bacteria within a Hot Spring Microbial Mat (Nakabusa Hot Springs, Japan). Microbes Environ..

[13] Burgess E.A., Unrine J.M., Mills G.L., Romanek C.S., Wiegel J. (2012). Comparative Geochemical and Microbiological Characterization of Two Thermal Pools in the Uzon Caldera, Kamchatka, Russia. Microb. Ecol..

[14] Rozanov A.S., Bryanskaya A.V., Malup T.K., Meshcheryakova I.A., Lazareva E.V., Taran O.P., Ivanisenko T.V., Ivanisenko V.A., Zhmodik S.M., Kolchanov N.A. (2014). Molecular analysis of the benthos microbial community in Zavarzin thermal spring (Uzon Caldera, Kamchatka, Russia). BMC Genom..

[15] Merkel A.Y., Pimenov N.V., Rusanov I.I., Slobodkin A.I., Slobodkina G.B., Tarnovetckii I.Y., Frolov E.N., Dubin A.V., Perevalova A.A., Bonch-Osmolovskaya E.A. (2017). Microbial diversity and autotrophic activity in Kamchatka hot springs. Extremophiles.

[16] Podosokorskaya O.A., Merkel A.Y., Kolganova T.V., Chernyh N.A., Miroshnichenko M.L., Bonch-Osmolovskaya E.A., Kublanov I.V. (2011). Fervidobacterium riparium sp. nov., a thermophilic anaerobic cellulolytic bacterium isolated from a hot spring. Int. J. Syst. Evol. Microbiol..

[17] Sokolova T.G., Kostrikina N.A., Chernyh N.A., Tourova T.P., Kolganova T.V., Bonch-Osmolovskaya E.A. (2002). Carboxydocella thermautotrophica gen. nov., sp. nov., a novel anaerobic, CO-utilizing thermophile from a Kamchatkan hot spring. Int. J. Syst. Evol. Microbiol..

[18] Andrews S., Krueger F., Seconds-Pichon A., Biggins F., Wingett S., FastQC (2015). A quality control tool for high throughput sequence data. Babraham Bioinformatics. Babraham Inst..

[19] Bolger A.M., Lohse M., Usadel B. (2014). Trimmomatic: A flexible trimmer for Illumina sequence data. Bioinformatics.

[20] Bankevich A., Nurk S., Antipov D., Gurevich A.A., Dvorkin M., Kulikov A.S., Lesin V.M., Nikolenko S.I., Pham S., Prjibelski A.D. (2012). SPAdes: A New Genome Assembly Algorithm and Its Applications to Single-Cell Sequencing. J. Comput. Biol..

[21] Mikheenko A., Saveliev V., Gurevich A. (2016). MetaQUAST: Evaluation of metagenome assemblies. Bioinformatics.

[22] Uritskiy G.V., DiRuggiero J., Taylor J. (2018). MetaWRAP—A flexible pipeline for genome-resolved metagenomic data analysis. Microbiome.

[23] Kang D.D., Froula J., Egan R., Wang Z. (2015). MetaBAT, an efficient tool for accurately reconstructing single genomes from complex microbial communities. PeerJ.

[24] Alneberg J., Bjarnason B.S., de Bruijn I., Schirmer M., Quick J., Ijaz U.Z., Loman N.J., Andersson A.F., Quince C. (2013). CONCOCT: Clustering cONtigs on COverage and ComposiTion. arXiv.

[25] Wu Y.-W., Simmons B.A., Singer S.W. (2016). MaxBin 2.0: An automated binning algorithm to recover genomes from multiple metagenomic datasets. Bioinformatics.

[26] Parks D.H., Imelfort M., Skennerton C.T., Hugenholtz P., Tyson G.W. (2015). CheckM: Assessing the quality of microbial genomes recovered from isolates, single cells, and metagenomes. Genome Res..

[27] Patro R., Duggal G., Love M.I., Irizarry R.A., Kingsford C. (2017). Salmon provides fast and bias-aware quantification of transcript expression. Nat. Methods.

[28] Rho M., Tang H., Ye Y. (2010). FragGeneScan: Predicting genes in short and error-prone reads. Nucleic Acids Res..

[29] Eddy S.R. (2011). Accelerated Profile HMM Searches. PLoS Comput. Biol..

[30] Segata N., Börnigen D., Morgan X.C., Huttenhower C. (2013). PhyloPhlAn is a new method for improved phylogenetic and taxonomic placement of microbes. Nat. Commun..

[31] Asnicar F., Thomas A.M., Beghini F., Mengoni C., Manara S., Manghi P., Zhu Q., Bolzan M., Cumbo F., May U. (2020). Precise phylogenetic analysis of microbial isolates and genomes from metagenomes using PhyloPhlAn 3.0. Nat. Commun..

[32] Asnicar F., Weingart G., Tickle T.L., Huttenhower C., Segata N. (2015). Compact graphical representation of phylogenetic data and metadata with GraPhlAn. PeerJ.

[33] Mukherjee S., Seshadri R., Varghese N.J., Eloe-Fadrosh E.A., Meier-Kolthoff J.P., Göker M., Coates R.C., Hadjithomas M., Pavlopoulos G.A., Paez-Espino D. (2017). 1,003 reference genomes of bacterial and archaeal isolates expand coverage of the tree of life. Nat. Biotechnol..

[34] Gruber-Vodicka H.R., Seah B.K.B., Pruesse E. (2019). phyloFlash—Rapid SSU rRNA profiling and targeted assembly from metagenomes. bioRxiv.

[35] Kumar S., Stecher G., Li M., Knyaz C., Tamura K. (2018). MEGA X: Molecular Evolutionary Genetics Analysis across Computing Platforms. Mol. Biol. Evol..

[36] Kyrpides R.M., Stepanauskas N.C. (2017). Minimum information about a single amplified genome (MISAG) and a metagenome-assembled genome (MIMAG) of bacteria and archaea. Nat. Biotechnol..

[37] De Luca P., Musacchio A., Taddei R. (1981). Acidophilic algae from the fumaroles of mount lawu (Java, locus classicus of cyanidium caldarium geitler. G. Bot. Ital..

[38] Enami I., Adachi H., Shen J.-R. (2010). Mechanisms of Acido-Tolerance and Characteristics of Photosystems in an Acidophilic and Thermophilic Red Alga, Cyanidium Caldarium. Red Algae in the Genomic Age.

[39] Ott F.D., Seckbach J. (1994). A review on the taxonomic position of the algal genus Cyanidium Geitler 1933 and its ecological cohorts Galdieria Merola in Merola et al. 1981 and Cyanidioschyzon De Luca, Taddei and Varano 1978. Evolutionary Pathways and Enigmatic Algae: Cyanidium Caldarium (Rhodophyta) and Related Cells.

[40] Golyshina O.V., Kublanov I.V., Tran H., Korzhenkov A.A., Lünsdorf H., Nechitaylo T.Y., Gavrilov S.N., Toshchakov S.V., Golyshin P.N. (2016). Biology of archaea from a novel family Cuniculiplasmataceae (Thermoplasmata) ubiquitous in hyperacidic environments. Sci. Rep..

[41] O’Malley M.A. (2008). ‘Everything is everywhere: But the environment selects’: Ubiquitous distribution and ecological determinism in microbial biogeography. Stud. Hist. Philos. Sci. Part C.

